# The clinical relevance of omega-3 fatty acids in the management of hypertriglyceridemia

**DOI:** 10.1186/s12944-016-0286-4

**Published:** 2016-07-22

**Authors:** James Backes, Deborah Anzalone, Daniel Hilleman, Julia Catini

**Affiliations:** Atherosclerosis and LDL-Apheresis Center, School of Pharmacy, University of Kansas, 3901 Rainbow Boulevard, Kansas City, KS 66160 USA; AstraZeneca, Wilmington, DE USA; Creighton University, Omaha, NE USA

**Keywords:** Docosahexaenoic acid, Docosapentaenoic acid, Eicosapentaenoic acid, Hypertriglyceridemia, Omega-3 fatty acids

## Abstract

Hypertriglyceridemia (triglycerides > 150 mg/dL) affects ~25 % of the United States (US) population and is associated with increased cardiovascular risk. Severe hypertriglyceridemia (≥ 500 mg/dL) is also a risk factor for pancreatitis. Three omega-3 fatty acid (OM3FA) prescription formulations are approved in the US for the treatment of adults with severe hypertriglyceridemia: (1) OM3FA ethyl esters (OM3EE), a mixture of OM3FA ethyl esters, primarily eicosapentaenoic acid (EPA) and docosahexaenoic acid (DHA) (Lovaza®, Omtryg™, and generics); (2) icosapent ethyl (IPE), EPA ethyl esters (Vascepa®); and (3) omega-3 carboxylic acids (OM3CA), a mixture of OM3FAs in free fatty acid form, primarily EPA, DHA, and docosapentaenoic acid (Epanova®). At approved doses, all formulations substantially reduce triglyceride and very-low-density lipoprotein levels. DHA-containing formulations may also increase low-density lipoprotein cholesterol. However, this is not accompanied by increased non-high-density lipoprotein cholesterol, which is thought to provide a better indication of cardiovascular risk in this patient population. Proposed mechanisms of action of OM3FAs include inhibition of diacylglycerol acyltransferase, increased plasma lipoprotein lipase activity, decreased hepatic lipogenesis, and increased hepatic β-oxidation. OM3CA bioavailability (area under the plasma concentration-time curve from zero to the last measurable concentration) is up to 4-fold greater than that of OM3FA ethyl esters, and unlike ethyl esters, the absorption of OM3CA is not dependent on pancreatic lipase hydrolysis. All three formulations are well tolerated (the most common adverse events are gastrointestinal) and demonstrate a lack of drug-drug interactions with other lipid-lowering drugs, such as statins and fibrates. OM3FAs appear to be an effective treatment option for patients with severe hypertriglyceridemia.

## Background

Hypertriglyceridemia, defined as a triglyceride (TG) level of > 150 mg/dL, affects approximately 25 % of the United States (US) population [1]. High levels of TG (≥ 200 mg/dL) have been shown to be independently associated with an increased risk of cardiovascular disease (CVD), even in patients treated effectively with statins to reduce low-density lipoprotein cholesterol (LDL-C) [2, 3]. With increased TG, there are elevations of TG-rich lipoproteins (TRL) (very-low-density lipoproteins [VLDL] plus chylomicrons) and their remnants which have been shown to contribute to the progression of atherosclerosis and CVD via a number of direct and indirect mechanisms. These mechanisms include the direct contribution to intimal cholesterol deposition, and the activation and enhancement of pro-inflammatory, pro-apoptotic, and pro-coagulant pathways [4].

Severe hypertriglyceridemia, defined by the 2014 National Lipid Association (NLA) guidelines as a TG level of ≥ 500 mg/dL [5], is also a well-known risk factor for pancreatitis, reportedly causing up to 7 % of cases [6]. Severe hypertriglyceridemia usually occurs as a result of one or more genetic disorders, such as hyperlipoproteinemia or lipoprotein lipase (LPL) deficiency [7]. In this instance, elevated levels of chylomicrons are thought to obstruct capillaries in the pancreas, leading to local ischemia, edema, and inflammation [6].

Consequently, it is important that patients with hypertriglyceridemia receive safe and effective treatment to reduce the risk of pancreatitis and CVD. In severe hypertriglyceridemia, guidelines advocate the immediate use of TG-lowering drugs [5, 8], which include fibrates, niacin, and omega-3 fatty acids (OM3FAs). However, in patients with moderately elevated TG, statins are often recommended as first-line therapy. Although statins primarily target elevated LDL-C, in patients with high baseline TG levels (≥ 273 mg/dL), some high-intensity statins have been shown to reduce TG by up to 43 % [9]. That said, if elevated TG or non-high-density lipoprotein cholesterol (non-HDL-C) levels persist following lifestyle intervention and statin therapy, a number of guidelines recommend the addition of a TG-lowering drug [5]. Although all of these drugs successfully lower TG levels, the high incidence of adverse events associated with niacin therapy and the increased incidence of myopathy and rhabdomyolysis observed with some fibrate-statin combinations have raised concerns [10, 11]. In April 2016, the Food and Drug Administration (FDA) concluded that the benefits of the concomitant use of statins plus extended release niacin or some fibrates do not outweigh the risks, and have, therefore, withdrawn the approvals for this indication [12]. However, the indications for the treatment of severe hypertriglyceridemia remain intact. It has been suggested that OM3FAs may be a well-tolerated and effective alternative to fibrates and niacin in the treatment and management of hypertriglyceridemia [13].

Prescription formulations of OM3FAs are indicated in the US as an adjunct to diet to reduce TG levels in adults with severe hypertriglyceridemia. There are three prescription OM3FA formulations approved in the US: (1) omega-3 fatty acid ethyl esters (OM3EE), a mixture of long-chain omega-3 fatty acid ethyl esters, primarily eicosapentaenoic acid (EPA) and docosahexaenoic acid (DHA) (Lovaza®, Omtryg™, and some generics) [14, 15]; (2) icosapent ethyl (IPE), EPA ethyl esters (Vascepa®) [16]; (3) and omega-3 carboxylic acids (OM3CA), a mixture of long-chain OM3FAs in free fatty acid form, primarily EPA, DHA, and docosapentaenoic acid (DPA) (Epanova®) [17]. Of note, although Epanova and Omtryg have been approved in the US by the FDA, and Epanova is currently being studied in the prevention of adverse cardiovascular outcomes, they are not yet commercially available. Although these prescription formulations have all been shown to significantly reduce TG levels in patients with high or very high TG levels, and they appear to be generally well tolerated in these patient populations, they differ in their effects on individual lipid parameters and bioavailability [18–24]. OM3FA dietary supplements are also available, but as these products do not need to comply with the rigorous regulations required for prescription formulations, and do not require approval from the FDA, their OM3FA content is often inconsistent and may be inadequate to effectively treat hypertriglyceridemia [25–27].

This review aims to evaluate the clinical relevance of OM3FAs in the management of hypertriglyceridemia and to explore the pharmacological and mechanistic properties, as well as the efficacy and safety, of three prescription OM3FA formulations.

### Search strategy

A search of PubMed was performed using the following search strategy: (“Hypertriglyceridemia”[MeSH]) AND “Fatty Acids, Omega-3”[MeSH]).

The search was limited to English-language, human-only studies published from August 17, 2005, to August 17, 2015. The literature search yielded a total of 166 publications. The reference lists of articles identified using this search strategy were also searched, meaning that widely referenced, older publications were also screened. Articles were deemed eligible for inclusion if they provided useful and clinically relevant information pertaining to prescription formulations of OM3FAs, or the treatment and management of hypertriglyceridemia. In addition, the US prescribing information for prescription OM3FAs and current guidelines on the treatment and management of dyslipidemia were reviewed.

### Management of hypertriglyceridemia

The clinical definitions of hypertriglyceridemia, as outlined in the 2014 NLA guideline on the management of dyslipidemia, are shown in Table 1. However, it is important to note that the precise clinical definitions of the severity of hypertriglyceridemia differ among guidelines. For example, although severe hypertriglyceridemia is typically classified as a TG level ≥ 500 mg/dL [5, 28–30], the 2012 Endocrine Society guideline and the 2014 American Diabetes Association guideline classify severe hypertriglyceridemia as a TG level ≥ 1000 mg/dL [31, 32]. The majority of guidelines agree that a TG level of < 150 mg/dL is desirable [5, 28–31, 33, 34].

**Table 1:** Clinical definition of hypertriglyceridemia [5]

Category	TG, mg/dL (mmol/L)
Normal	< 150 (< 1.7)
Borderline-high	150–199 (1.7–2.2)
High	200–499 (2.2–5.6)
Very high (severe HTG)	≥ 500 (≥ 5.6)

*HTG* hypertriglyceridemia, *TG* triglyceride

Although current guidelines advocate the immediate use of a TG-lowering drug in patients with very high TG levels (≥ 500 mg/dL) and recommend that lifestyle intervention and statin treatment should be considered as first-line therapy in patients with moderately elevated TG, they can vary in some aspects of their treatment and management recommendations [5, 28–31, 33, 34]. The 2014 NLA guideline recommends using LDL-C or non-HDL-C as the primary treatment target in patients with dyslipidemia. They also emphasize that as non-HDL-C comprises all the cholesterol carried by potentially atherogenic lipoprotein particles, it is a stronger predictor of CVD than LDL-C. In addition, they consider apolipoprotein B (ApoB) as an optional secondary treatment target [5]. This guidance is in contrast to the 2013 American College of Cardiology (ACC)/American Heart Association (AHA) guideline, which focuses on the use of fixed-dose, moderate- to high-intensity statin therapy to reduce LDL-C levels in patients at high CVD risk [8].

Like most guidelines, neither the NLA nor ACC/AHA guideline considers elevated TG to be a treatment target unless levels are very high (≥ 500 mg/dL). The NLA guideline highlights that in this case, TG lowering becomes the primary management goal, and the immediate use of a TG-lowering drug, including high-dose OM3FAs, is warranted [5]. Although the ACC/AHA guideline does not make any specific recommendations for the treatment of patients with very high TG levels, it does direct the reader to the 2011 AHA scientific statement on TG and CVD, which recommends the use of pharmacological therapy with a TG-lowering drug, including fibrates, niacin, or OM3FAs [28].

In the management of elevated TG levels, both the 2014 NLA guideline and the 2011 AHA scientific statement focus on intensive therapeutic lifestyle intervention, including a 5–10 % reduction in body weight, restriction of alcohol and sugar intake, and increased physical activity [5, 28]. The NLA guideline also states that when TG levels are high (200–499 mg/dL), non-HDL-C remains the primary treatment target, and statins the first-line therapy choice. However, if non-HDL-C treatment goals (< 130 mg/dL for low- to high-risk patients or < 100 mg/dL for very-high-risk patients) are not achieved with the maximum tolerated statin therapy, the guideline advocates the addition of a TG-lowering drug, such as OM3FAs, fibrates, or niacin, to statin therapy in patients with hypertriglyceridemia [5].

Like the 2014 NLA guideline, a number of other guidelines pertaining to the treatment and management of hypertriglyceridemia also recommend the use of TG-lowering drugs (including high-dose OM3FAs), alone or in combination with statin therapy, in patients who continue to have elevated TG or non-HDL-C levels despite being at LDL-C treatment goal [31, 33, 34]. That being said, as mentioned previously, in 2016 the FDA withdrew the approval for extended release niacin and some fibrates when coadministered with statins [12].

### Mechanism of action of omega-3 fatty acids

**Fig. 1:**
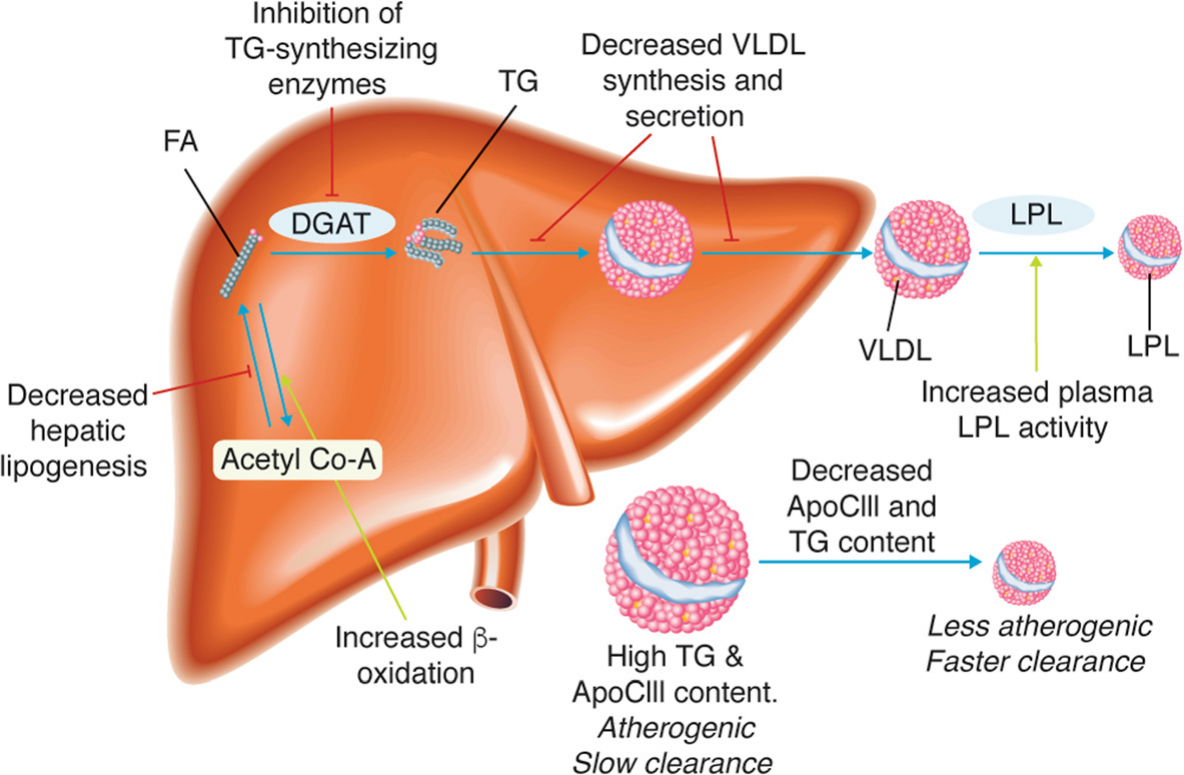
Proposed mechanisms of action of prescription formulations of long-chain omega-3 fatty acids. *ApoCIII* apolipoprotein CIII, *Acetyl Co-A* acetyl coenzyme A, *DGAT* diglyceride acyltransferase; *FA* fatty acid, *LPL* lipoprotein lipase, *TG* triglyceride, *VLDL* very-low-density lipoprotein

Although the TG-lowering ability of prescription OM3FAs is well established, the exact TG-lowering mechanisms of action are not completely understood. Results from preclinical and clinical studies suggest that OM3FAs decrease serum TG concentrations by reducing TG synthesis, reducing the incorporation of TG into VLDL, reducing TG secretion, and enhancing TG clearance from VLDL particles [35] (Fig. 1). It has been proposed that OM3FAs exert these TG-lowering effects via a number of mechanisms: (1) OM3FAs are thought to decrease hepatic lipogenesis by suppressing the expression of sterol regulatory element-binding protein-1c. This, in turn, leads to decreased expression of cholesterol-, fatty acid-, and TG-synthesizing enzymes [36, 37]. (2) They are thought to increase the β-oxidation of fatty acids, resulting in a reduction in available substrate required for TG and VLDL synthesis [35]. (3) They are thought to inhibit key enzymes involved in hepatic TG synthesis, such as phosphatidic acid phosphatase and diacylglycerol acyltransferase [38]. (4) Finally, they have been shown to increase the expression of LPL, a key component of the TRL biosynthetic pathways, leading to increased TG removal from circulating VLDL and chylomicron particles [39, 40].

The primary components of OM3FA prescription formulations, EPA and DHA, have both been shown to reduce TG. However, they are known to have differing effects on LDL-C and HDL-C [41–43]. In a meta-analysis comparing the effects of DHA and EPA, in direct comparison studies, DHA was associated with a greater reduction in TG and a greater increase in LDL-C than EPA. DHA also raised HDL-C compared with placebo, whereas EPA did not [42]. Further research is needed to elucidate the mechanisms and significance of these differences [41–43].

OM3CAs have also been shown to reduce levels of apolipoprotein CIII (ApoCIII) [44]. ApoCIII is thought to contribute to the progression of atherosclerosis and CVD via a number of mechanisms, including the activation and enhancement of pro-inflammatory pathways [45]. It also inhibits the receptor-mediated uptake of TRL and their remnants, slowing their clearance rate, and promotes the formation of small dense LDL particles from VLDL [44, 45]. Additionally, ApoCIII is a key contributor to hypertriglyceridemia, primarily due to its inhibitory actions on LPL. LPL hydrolyzes plasma TRL, producing free fatty acids, chylomicron remnants and intermediate-density lipoproteins (IDL). Some IDL particles can also undergo further LPL-mediated hydrolysis to produce LDL [28]. It has been hypothesized that the differing effects of DHA and EPA on the lipid profile may be due to their differentiating effects on ApoCIII synthesis. DHA is thought to reduce ApoCIII synthesis by regulating a number of hepatic transcription factors, such as hepatic nuclear factor-4-alpha and forkhead box-O transcription factor O1. Therefore, DHA enhances the hydrolysis of VLDL, resulting in greater conversion to LDL, and the formation of larger, more buoyant LDL particles [43]. However, these effects are uncertain and warrant additional investigation.

In addition to their TG-lowering effects, OM3FA are thought to act on additional cardiometabolic risk factors, and have been shown to significantly decrease a number of inflammatory markers associated with atherosclerosis and CVD. Clinical studies have demonstrated that OM3CA and IPE decrease levels of lipoprotein-associated phospholipase A2 (Lp-PLA2) versus placebo, and IPE has been shown to decrease high-sensitivity C-reactive protein [21, 23, 46]. Pre-clinical and clinical studies have also found that EPA and DHA exhibit antiarrhythmic and antioxidant effects [47, 48], improve endothelial function [49, 50], and promote a less atherogenic lipoprotein subfraction profile when administered in addition to statin therapy [51]. They have also been shown to decrease platelet activity biomarkers in comparison with placebo, regardless of concomitant aspirin and statin therapy use, and it is believed that they decrease platelet aggregation [52]. However, DHA is thought to display more potent anti-aggregatory effects than EPA [49, 50, 53]. In addition, DHA has been shown to lower blood pressure and heart rate [54–56], and seems to have a protective effect on cognitive decline [57]. Other studies have shown an inverse association between carotid intima-media thickness and OM3FA use, suggesting that OM3FA may protect against carotid atherosclerosis [58], with data indicating a decrease in plaque inflammation and an increase in plaque stability [59]. Despite these proposed cardioprotective mechanisms, to date, the effects of OM3FA-mediated TG lowering on cardiovascular outcomes and the incidence of pancreatitis have not yet been determined in large-scale clinical trials [14, 16, 17, 60].

Although the majority of research on the therapeutic potential of OM3FAs has focused on EPA and DHA, emerging research has also begun to elucidate the mechanisms of DPA. DPA levels are independently associated with a decreased risk of myocardial infarction and coronary heart disease [61, 62], and low levels of DPA have also been shown to be associated with lipid-rich plaques and peripheral arterial disease [63, 64]. Studies have demonstrated that EPA, DHA, and DPA dose-dependently inhibit platelet aggregation. However, DPA was shown to be a more potent platelet inhibitor than EPA and DHA [65]. Like DHA and EPA, DPA has also been shown to reduce the expression of inflammatory genes [66].

As the TG-lowering mechanism of action of long-chain OM3FAs differs from that of other lipid-lowering drugs, such as statins, they can potentially provide complementary benefits on the lipid profile when administered in combination [35]. This is corroborated by the fact that the level of TG lowering achieved with OM3FAs provides incremental reductions in TG levels when added to statin therapy [20, 22, 24, 67].

### Bioavailability

Absorption of DHA and EPA has been shown to vary between formulations. OM3CA have a 4-fold greater bioavailability (area under the plasma concentration-time curve from zero to the last measurable concentration [AUC_(0-t)_]) for both EPA and DHA during low fat-consumption periods, compared with that of OM3FA ethyl esters [68]. The absorption of OM3FA ethyl esters (OM3EE and IPE) requires hydrolysis with pancreatic lipase [69, 70], and as pancreatic lipase levels are dependent on the quantity and the type of lipids ingested, the absorption of OM3FA ethyl esters is thought to be highly dependent on meal fat content [71, 72]. In contrast, OM3FAs in the free fatty acid form, found in OM3CA, are not dependent on pancreatic lipase hydrolysis. Therefore, the bioavailability of EPA and DHA from this formulation is less dependent on meal fat content than OM3FA ethyl esters [68]. In one study (*n* = 54), under low-fat conditions, 59 % of subjects dosed with OM3CA maintained an AUC_(0-t)_ for EPA and DHA of ≥50 % of the respective AUC_(0-t)_ observed in high-fat conditions, compared with only 6 % of subjects dosed with OM3EE. This may confer a potential therapeutic advantage, as current guidelines recommend that patients with severe hypertriglyceridemia follow a very-low-fat diet [5, 28]. This increased bioavailability, particularly during low fat consumption, may also allow greater flexibility with regard to dosing schedule. OM3CA have been approved to effectively lower TG levels at doses of 2 g/day or 4 g/day, in a once-daily dosing regimen without regard to meals [22].

### Efficacy of omega-3 fatty acid formulations

**Fig. 2:**
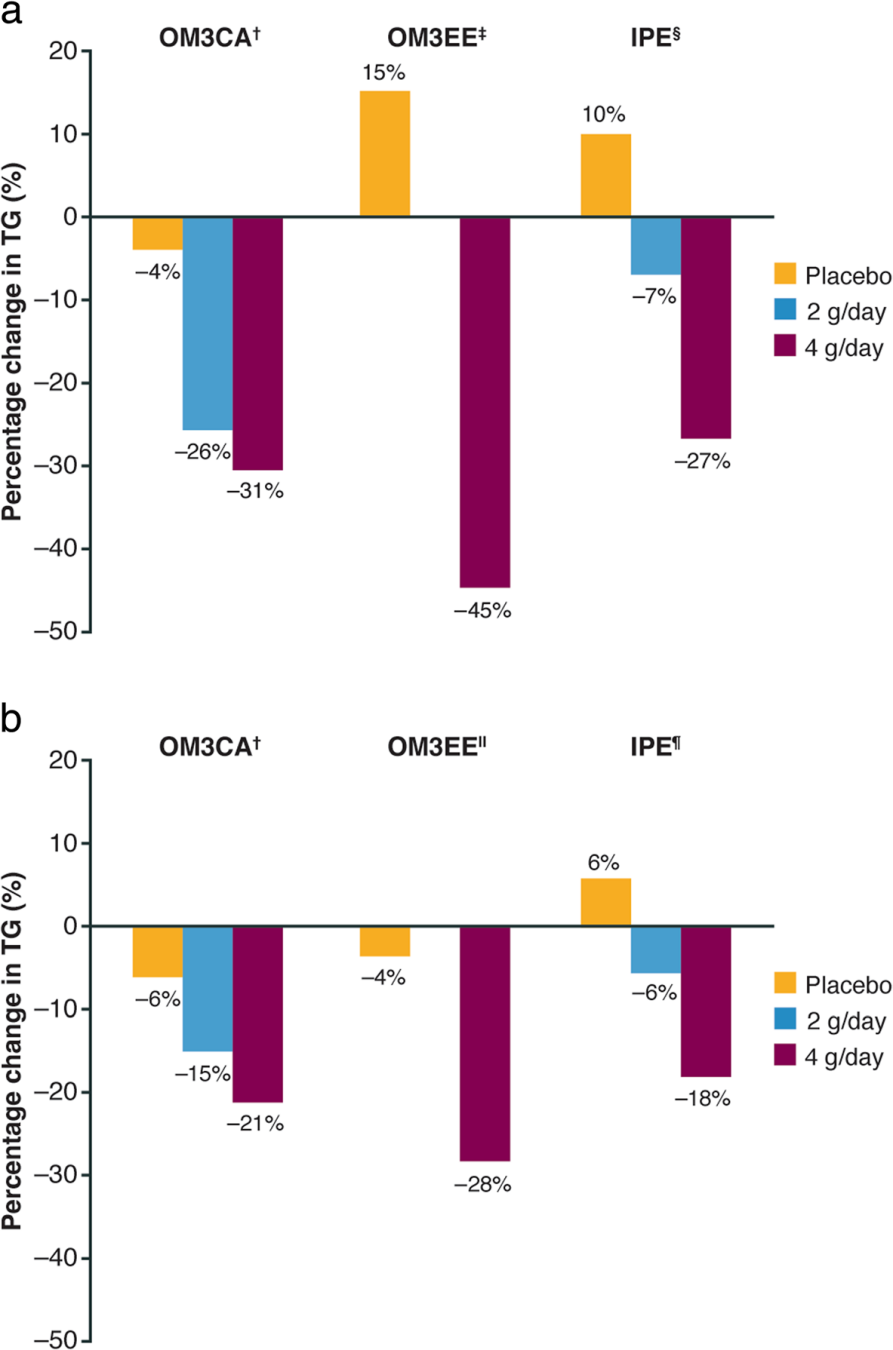
TG reduction observed with varying doses of omega-3 carboxylic acids, omega-3 ethyl esters, and icosapent ethyl in (**a**) patients with severe hypertriglyceridemia (TG ≥ 500 mg/dL) [14, 16, 17] and (**b**) statin-treated patients with high baseline TG levels (TG level ≥ 200 mg/dL and < 500 mg/dL) [20, 21, 24] Data not available for OM3EE at 2 g/day. OM3CA = Percentage change from baseline expressed as least-squares geometric mean; OM3EE = percentage change from baseline expressed as geometric mean; IPE = percentage change from baseline expressed as median. ^†^Placebo used = olive oil; ^‡^Placebo used = vegetable oil; ^§^Placebo used = mineral oil; ^ǁ^Placebo used = corn oil; ^¶^Placebo not specified. *IPE* icosapent ethyl, *OM3CA* omega-3 carboxylic acids, *OM3EE* omega-3 ethyl esters, *TG* triglyceride

The effects of the three OM3FA prescription formulations on lipid parameters in patients with severe hypertriglyceridemia are summarized in Table 2. These data are reported in the US prescribing information for OM3EE (Lovaza) [14], IPE (Vascepa) [16], and OM3CA (Epanova) [17], which each summarize the results from the respective pivotal, randomized, double-blind, placebo-controlled trials conducted in patients with baseline TG levels of 500–2000 mg/dL [18, 19, 21, 23]. It is important to note that the type of placebo used differs among these clinical trials, and each placebo has differing effects on TG. The olive oil comparator used in the OM3CA trial substantially decreased TG levels, whereas the corn oil and mineral oil used in the OM3EE and IPE trials increased TG levels. In patients with TG levels > 500 mg/dL, the TG reductions observed with all prescription formulations, at doses of 4 g/day, were statistically significant compared with placebo, thus allowing the US FDA to approve these formulations for adults with severe hypertriglyceridemia. As might be expected, higher OM3FA doses and higher baseline TG levels are associated with greater percentage TG reductions [73] (Fig. 2). Reductions in VLDL-C levels versus placebo were also shown to be statistically significant.

**Table 2:** Change in lipid parameters observed with omega-3 carboxylic acids, omega-3 ethyl esters, and icosapent ethyl in patients with severe hypertriglyceridemia (triglyceride level ≥ 500 mg/dL)

	OM3CA (Epanova) [17]			OM3EE (Lovaza) [14]		IPE (Vascepa) [16]	
Parameter	Placebo (olive oil) (*n* = 100)	OM3CA 2 g/day (*n* = 100)	OM3CA 4 g/day (*n* = 99)	Placebo (corn oil) (*n* = 42)	OM3EE 4 g/day (*n* = 42)	Placebo (mineral oil) (*n* = 75)	IPE 4 g/day (*n* = 76)
TG							
Median BL, mg/dL	682	717	655	788	816	703	680
Median percentage change from BL, %	−10	−25	−31	7	−45	10	−27
Difference^‡^	–	−16**	−21***	–	−52	–	−33***
Non-HDL-C							
Median BL, mg/dL	215	205	225	292	271	229	225
Median percentage change from BL, %	−1	−8	−8	−4	−14	8	−8
Difference^‡^	–	−7*	−10**	–	−10	–	−18
HDL-C							
Median BL, mg/dL	29	27	29	24	22	27	27
Median percentage change form BL, %	2	7	5	0	9	0	−4
Difference^‡^	–	5^†^	4^†^	–	9	–	−4
Total cholesterol							
Median BL, mg/dL	246	241	254	314	296	256	254
Median percentage change from BL, %	0	−6	−6	−2	−10	8	−7
Difference^‡^	–	−6	−9	–	−8	–	−16
VLDL-C							
Median BL, mg/dL	125	123	126	175	175	124	123
Median percentage change from BL, %	−11	−25	−35	−1	−42	14	−20
Difference^‡^	–	−14	−21	–	−41	–	−29*
LDL-C							
Median BL, mg/dL	78	77	90	108	89	86	91
Median percentage change from BL, %	10	21	26	−5	45	−3	−5
Difference^‡^	–	13	15	–	49	–	−2
ApoB							
Median BL, mg/dL	110	114	118	ND	ND	118	121
Median percentage change from BL, %	2	6	6	ND	ND	4	−4
Difference^‡^	–	3	2	–	ND	–	−9*

*P*-values from Wilcoxon rank sum test: *P < 0.05; **P < 0.01; ***P < 0.001; ^†^not significant. Testing for statistical significance was performed for OM3CA (TG, non-HDL-C and HDL-C) and IPE (TG, VLDL-C, and ApoB) only. No statistical analysis was presented for OM3EE

^‡^Difference for OM3CA and IPE = median of [omega-3 fatty acid formulation % change – placebo % change] (Hodges-Lehmann Estimate); Difference for OM3EE = OM3EE median % change – placebo median % change

The median placebo-corrected % change in lipid parameters reported for Omtryg, the generic form of Lovaza, are as follows: TG = −12 %, non-HDL-C = −9 %, HDL-C = 4 %, TC = −7 %, VLDL-C = −29 %, and LDL-C = 25 % [15]

*ApoB* apolipoprotein B, *BL* baseline, *HDL-C* high-density lipoprotein cholesterol, *IPE* icosapent ethyl, *LDL-C* low-density lipoprotein cholesterol, *ND* not described, *OM3CA* omega-3 carboxylic acids, *OM3EE* omega-3 ethyl esters, *TG* triglyceride, *VLDL-C* very-low-density lipoprotein cholesterol

It should be noted that there appears to be a tendency for DHA-containing formulations (OM3CA and OM3EE) to increase LDL-C versus baseline. However, importantly, this is not associated with an accompanying increase in non-HDL-C or ApoB, and as mentioned earlier, non-HDL-C levels are thought to provide a better indication of CVD risk than LDL-C levels. This is particularly the case for patients with hypertriglyceridemia, who often have low levels of LDL-C [5, 34]. Additionally, unlike IPE, DHA-containing formulations increase HDL-C levels. However, the clinical relevance of increasing HDL-C using any pharmacologic agent remains unclear.

Similar effects on lipid parameters are observed in statin-treated patients with high TG levels (≥ 200 mg/dL and < 500 mg/dL) (Table 3) [20, 22, 24]. All prescription formulations of OM3FAs at a dose of 4 g/day were shown to significantly reduce TG, VLDL-C, non-HDL-C, and ApoB levels in this patient population. However, in contrast to patients with severe hypertriglyceridemia (TG ≥ 500 mg/dL), no increase in LDL-C was observed with any of the formulations. This is likely due to the fact that these patients had lower baseline TG levels and were also receiving statin therapy. The levels of TG reduction observed with varying doses of the three OM3FA formulations in patients with severe hypertriglyceridemia and high levels of TG are shown in Fig. 2a and b.

**Table 3:** Change in lipid parameters observed with omega-3 carboxylic acids, omega-3 ethyl esters, and icosapent ethyl in statin-treated patients with high triglyceride levels (triglyceride level ≥ 200 mg/dL and < 500 mg/dL)

	OM3CA (Epanova) [22]			OM3EE (Lovaza) [20]		IPE (Vascepa) [24]		
Parameter	Statin^†^ + olive oil (*n* = 215)	Statin^†^ + OM3CA 2 g/day (*n* = 215)	Statin^†^ + OM3CA 4 g/day (*n* = 215)	Simvastatin 40 mg + vegetable oil (*n* = 132)	Simvastatin 40 mg + OM3EE 4 g/day (*n* = 122)	Statin^‡^ + placebo^§^ (*n* = 277)	Statin^‡^ + IPE 2 g/day (*n* = 234)	Statin^‡^ + IPE 4 g/day (*n* = 226)
TG								
BL, mg/dL	280	284	287	286.7	282.0	259.0	254	264.8
% change	−5.9	−14.6***	−20.6***	−3.5	−28.2***	5.9	−5.6**	−17.5***
Non-HDL-C								
BL, mg/dL	135	140	139	141.3	135.8	128	128	128
% change	−0.9	−3.9*	−6.9***	−1.5	−7.9***	9.8	2.4	−5.0***
HDL-C								
BL, mg/dL	38.8	38.7	38.8	44.7	47.3	39.0	38.0	37.0
% change	2.2	2.6	3.3	−1.1	4.1***	4.8	0.0	−1.0**
Total cholesterol								
BL, mg/dL	174	179	178	186.0	183.1	168.0	169	167
% change	0.5	−1.7*	−3.8***	−1.5	−4.7**	9.1	2.1	−3.2***
VLDL-C								
BL, mg/dL	45.7	46.9	47.2	53.2	52.1	42.0	43.0	44.0
% change	−5.9	−14.3**	−21.5***	−4.8	−23.8***	15.0	1.6	−12.1***
LDL-C								
BL, mg/dL	91.7	92.3	93.6	92.3	89.2	84.0	82.0	82.0
% change	1.1	4.6*	1.3	−1.9	3.4	8.8	2.4	1.5**
ApoB								
BL, mg/dL	93.8	94.5	95.9	86.8	85.0	91.0	91.0	93.0
% change	0.3	0.7	−2.1*	−1.2	−3.8*	7.1	1.6	−2.2***

For IPE, data are presented as median; for OM3CA, data are presented as least-squares mean; for OM3EE, data are presented as geometric mean. *P*-values versus placebo: *P < 0.05; **P < 0.01; ***P < 0.001. ^†^Statins included lovastatin 20–40 mg, pravastatin 10–80 mg, fluvastatin 20–80 mg, simvastatin 10–40 mg, atorvastatin 10–40 mg, and rosuvastatin 10–40 mg, alone or in combination with ezetimibe; ^‡^Statins included simvastatin, atorvastatin, and rosuvastatin, alone or in combination with ezetimibe. ^§^Placebo not specified

*ApoB* apolipoprotein B, *BL* baseline, *HDL-C* high-density lipoprotein cholesterol, *IPE* icosapent ethyl, *LDL-C* low-density lipoprotein cholesterol, *OM3CA* omega-3 carboxylic acids, *OM3EE* omega-3 ethyl esters, *TG* triglyceride, *VLDL-C* very-low-density lipoprotein cholesterol

To date, the effect of any of the prescription OM3FA formulations on pancreatitis has not been determined. Furthermore, no large-scale, randomized clinical trials have provided clear evidence for the association between TG lowering and CVD risk reduction [14, 16, 17, 60]. Two large-scale, multinational cardiovascular outcomes studies (the Reduction of Cardiovascular Events with EPA – Intervention Trial [REDUCE-IT; NCT01492361] [74] and STatin Residual risk reduction with EpaNova in hiGh cardiovascular risk paTients with Hypertriglyceridemia [STRENGTH; NCT02104817] [75]) are currently under way to address this question. These trials aim to evaluate the reduction in residual risk of statin-treated patients with high TGs treated with OM3FA. The trials will examine the safety and efficacy of prescription strength long-chain OM3FAs in combination with statin therapy in high-risk patients with hypertriglyceridemia, defined as TG up to 500 mg/dL.

#### Omega-3 fatty acids in pediatric populations

The process of atherosclerosis can begin in early childhood, particularly in the presence of known cardiovascular risk factors, such as obesity, hypertension, and diabetes mellitus [76]. Studies have shown that omega-3 fatty acid supplementation can have beneficial effects on preclinical atherosclerosis markers in children with cardiovascular risk factors, including the prevention of carotid artery intima-media thickness and increased artery flow-mediated vasodilation [50, 77]. In addition, dietary omega-3 fatty acid supplementation in infancy has been associated with lower blood pressure in later childhood [78, 79]. Therefore, it has been suggested that dietary supplementation of omega-3 fatty acids in children may improve future cardiovascular outcomes [80].

### Safety

Prescription formulations of OM3FAs are generally well tolerated, with frequencies of treatment discontinuation similar between the OM3FA groups and placebo groups [81]. The most common adverse events associated with OM3FAs are gastrointestinal in nature (including eructation, nausea, diarrhea, and other mild gastrointestinal disturbances), and have been shown to occur in up to 27 % of patients at doses of 4 g/day [18, 21, 23]. The most common adverse events reported in the US prescribing information for each formulation are summarized in Table 4. Studies have shown that OM3FAs do not affect liver function [18], and do not have any known clinically significant drug–drug interactions with other commonly used lipid-modifying drugs, such as statins [82–84]. In contrast to a number of other TG-lowering drugs, studies have also shown that adverse events detected in patients receiving combination therapy with OM3FA ethyl esters and statins were similar to those detected in patients receiving statin monotherapy [20, 85, 86]. This is particularly advantageous when statin users with a history of common adverse events (e.g. myalgia, elevated hepatic transaminases) require combination therapy with a TG-lowering drug. Additionally, although OM3FAs have been shown to have the potential for antithrombotic effects, they have been shown not to increase clinical bleeding, even in patients receiving warfarin or antiplatelet drugs, including aspirin [87–90].

**Table 4:** Summary of adverse events observed with omega-3 carboxylic acids [17], omega-3 ethyl esters [14], and icosapent ethyl [16] in pooled placebo-controlled trials conducted in patients with hypertriglyceridemia, based on prescribing information for each formulation

Adverse event, %	OM3CA			OM3EE		IPE	
	Adverse events occurring at an incidence ≥ 3 %			Adverse events occurring at an incidence ≥ 3 %		Adverse events occurring at an incidence > 2 %	
	Placebo (olive oil) (*n* = 314)	OM3CA 2 g/day (*n* = 315)	OM3CA 4 g/day (*n* = 630)	Placebo (corn oil) (*n* = 370)	OM3EE (*n* = 655)	Placebo (mineral oil) (*n* = 309)	IPE (*n* = 622)
Diarrhea	2	7	15	–	–	–	–
Nausea	1	4	6	–	–	–	–
Abdominal pain or discomfort	2	3	5	–	–	–	–
Eructation	< 1	3	3	1	4	–	–
Dyspepsia	–	–	–	2	3	–	–
Taste perversion	–	–	–	< 1	4	–	–
Arthralgia	–	–	–	–	–	1	2

*IPE* icosapent ethyl, *OM3CA* omega-3 carboxylic acids, *OM3EE* omega-3 ethyl esters

It is not known whether patients with allergies to fish and/or shellfish are at an increased risk of allergic reaction to OM3FAs, and so these formulations should be used with caution in patients with known hypersensitivity to fish and/or shellfish [14, 16, 17].

### Dietary-supplement omega-3 fatty acids

Dietary-supplement OM3FAs are also widely utilized and are among the most popular dietary supplements worldwide [91]. However, dietary supplements are not subject to rigorous regulations required for prescription drugs. Consequently, the EPA and DHA content of dietary supplements may be inconsistent [25–27]. Due to an early evaluation study, practitioners typically view supplemental OM3FAs as adequate and reliable [92]. However, a more recent analysis of individual fish oil supplements found that they contain an inadequate dose of EPA and DHA. On average, they contained only 68 % of the claimed EPA and DHA content. The same analysis also found that the majority of supplements exceeded recommended levels of oxidation markers [26]. As the OM3FAs undergo oxidation, the concentration of EPA and DHA decreases, suggesting reduced efficacy. One study demonstrated that a median intake of 11 dietary fish oil supplement servings/day was required to achieve an FDA-approved dose of 3.4 g/day of OM3FAs [27, 93]. This study also found that dietary-supplement OM3FAs contain other fats and cholesterol, and that the fat and cholesterol content varies widely among products [27]. In addition, as the EPA and DHA content of dietary supplements varies among products, this may cause confusion for patients and practitioners. This is likely to result in inaccurate dosing that may potentially be inadequate to effectively lower TG in patients with hypertriglyceridemia.

Dietary omega-3 fatty acid intake has also been shown to play a role in cardiovascular risk reduction, with modest fish consumption (1–2 servings per week, particularly species high in EPA and DHA) associated with reduced risk of coronary death and total mortality [94]. Consequently, the AHA recommends including at least two servings of fish (preferably oily fish) per week [95]. In addition, it has been suggested that omega-3-enriched supplements (nutraceuticals) and functional foods (e.g. EPA- and DHA-enriched food products) may offer the possibility of increased EPA and DHA consumption [96, 97]. However, the benefits of nutraceutical products remain unclear, and robust clinical trials are required to establish their exact role in cardiovascular risk reduction [97].

### Conclusion

In severe hypertriglyceridemia (TG ≥ 500 mg/dL), guidelines advocate the immediate use of a TG-lowering drug to reduce the risk of pancreatitis. In patients with high TG levels (≥ 200 to < 500 mg/dL), some guidelines also recommend the use of TG-lowering drugs if elevated TG or non-HDL-C levels persist despite lifestyle intervention and statin therapy. Available TG-lowering drugs include fibrates, niacin, and OM3FAs. Unlike fibrates and niacin, OM3FAs do not have any known, clinically significant drug-drug interactions with other lipid-modifying therapies (such as statins) and do not affect liver function. In addition, as their mechanisms of action differ from those of other lipid-lowering drugs, they can potentially provide complementary benefits when administered as a combination therapy with statins.

All formulations have been shown to significantly reduce TG levels, and despite some differences in formulation, they appear to have similar efficacy. However, OM3FAs have been shown to differ in their effects on individual lipid parameters, their bioavailability, and the impact of fat content of meals on absorption. Although dietary supplements are available and widely utilized, they are not subject to the rigorous regulations required for prescription drugs, and therefore, their EPA and DHA content may be inconsistent and often may not meet label claims. All three prescription formulations of OM3FAs currently approved for use in the US appear to be generally well tolerated and effective in reducing TG levels in patients with hypertriglyceridemia.

## Abbreviations

ACC, American College of Cardiology; AHA, American Heart Association; Apo, apolioprotein; AUC_(0-t)_, area under the plasma concentration-time curve from zero to the last measurable concentration; CVD, cardiovascular disease; DHA, docosahexaenoic acid; DPA, docosapentaenoic acid; EPA, eicosapentaenoic acid; FDA, Food and Drug Administration; HDL-C, high-density lipoprotein cholesterol; IDL, intermediate-density lipoproteins; IPE, icosapent ethyl; LDL-C, low-density lipoprotein cholesterol; LPL, lipoprotein lipase; NLA, National Lipid Association; OM3CA, Omega-3 carboxylic acids; OM3EE, omega-3 fatty acid ethyl esters; OM3FA, omega-3 fatty acids; TG, triglyceride; TRL, TG-rich lipoproteins; US, United States; VLDL, very-low-density lipoproteins
